# Integrated profiling uncovers prognostic, immunological, and pharmacogenomic features of ferroptosis in triple-negative breast cancer

**DOI:** 10.3389/fimmu.2022.985861

**Published:** 2022-11-25

**Authors:** Kun Fang, Zhengjie Xu, Suxiao Jiang, Changsheng Yan, Desheng Tang, Yan Huang

**Affiliations:** ^1^ Department of Surgery, Yinchuan Maternal and Child Health Hospital, Yinchuan, China; ^2^ Department of Surgery, The First Affiliated Hospital of Harbin Medical University, Harbin, Heilongjiang, China; ^3^ Department of Surgery, Affiliated Hospital of Ningxia Medical University, Yinchuan, China

**Keywords:** triple-negative breast cancer, ferroptosis, prognosis, immunological feature, immunotherapy

## Abstract

**Objective:**

Ferroptosis is an iron-dependent type of regulated cell death triggered by the toxic buildup of lipid peroxides on cell membranes. Nonetheless, the implication of ferroptosis in triple‐negative breast cancer (TNBC), which is the most aggressive subtype of breast carcinoma, remains unexplored.

**Methods:**

Three TNBC cohorts—TCGA-TNBC, GSE58812, and METABRIC—were adopted. Consensus molecular subtyping on prognostic ferroptosis-related genes was implemented across TNBC. Ferroptosis classification-relevant genes were selected through weighted co-expression network analysis (WGCNA), and a ferroptosis-relevant scoring system was proposed through the LASSO approach. Prognostic and immunological traits, transcriptional and post-transcriptional modulation, therapeutic response, and prediction of potential small-molecule agents were conducted.

**Results:**

Three disparate ferroptosis patterns were identified across TNBC, with prognostic and immunological traits in each pattern. The ferroptosis-relevant scoring system was proposed, with poorer overall survival in high-risk patients. This risk score was strongly linked to transcriptional and post-transcriptional mechanisms. The high-risk group had a higher response to anti-PD-1 blockade or sunitinib, and the low-risk group had higher sensitivity to cisplatin. High relationships of risk score with immunological features were observed across pan-cancer. Two Cancer Therapeutics Response Portal (CTRP)-derived agents (SNX-2112 and brefeldin A) and PRISM-derived agents (MEK162, PD-0325901, PD-318088, Ro-4987655, and SAR131675) were predicted, which were intended for high-risk patients.

**Conclusion:**

Altogether, our findings unveil prognostic, immunological, and pharmacogenomic features of ferroptosis in TNBC, highlighting the potential clinical utility of ferroptosis in TNBC therapy.

## Introduction

Female breast cancer is the most commonly diagnosed cancer globally, with an estimated 2.3 million new cases (11.7%), and the major cause of cancer deaths with estimated 0.68 million deaths (6.9%) (1). Triple-negative breast cancer (TNBC) is a subtype of breast cancer with the absence of expression of estrogen receptor (ER) and progesterone receptor (PR) together with human epidermal growth factor receptor type 2 (HER2) (2). TNBC possesses a strong invasive and metastatic ability, easily invading blood vessels, and increased recurrence risk (2). The therapeutic options for TNBC are far more limited in comparison to those for other breast cancer subtypes (3). Surgical resection and chemotherapy remain the first‐line regimens against TNBC (3). Immunotherapy has revolutionized the field of oncology over the past few years, primarily with the introduction of immune checkpoint blockade (ICB) into clinical practice. Few patients with TNBC benefit from ICB, and complete and durable responses are rare because most tumors are not immunoreactive (4). Hence, an innovative scheme is required for bringing immunotherapy closer to TNBC. TNBC is highly genetically diverse, which ranges from high proliferation to chemotherapy resistance with low proliferative and luminal features (5). Biomarker selection, drug discovery, and clinical trial design are necessary to match properly targeted therapies to distinct subpopulations of TNBC patients.

Ferroptosis is an iron-dependent type of cell death induced by disruption of membrane integrity because of overproduced lipid peroxides, which is morphologically characterized by cell swelling, pore formation on the cell membrane, smaller mitochondria, and reduced mitochondrial cristae together with enhanced mitochondrial membrane density (6). Induction of ferroptotic cell death involves a few alterations (altered iron metabolism, response to oxidative stress, production of lipid peroxides, etc.) (7). Excessive or deficient ferroptosis correlates to various physiological and pathophysiological processes, especially dysregulated immune responses (8). For instance, CD8+ T cells trigger tumoral ferroptosis during cancer immunotherapy (9). Inducing ferroptosis may elicit an immunostimulatory tumor microenvironment (10). Due to the distinction of ferroptosis from apoptosis and others, inducing ferroptosis may eliminate tumor cells that have resistance to other cell death types (11). Ferroptotic cell death has become a novel direction around which to design TNBC therapy. Nonetheless, the full appearance of ferroptosis in TNBC has not yet been completely clarified.

The current study integrated ferroptosis-related genes (FRGs) and proposed a novel ferroptosis classification in TNBC. Especially, disparate ferroptosis patterns exhibited unique prognostic and immunological traits. Additionally, a ferroptosis-relevant gene signature was established and evaluated its associations with survival, immunological traits, and therapeutic sensitivity. Altogether, our findings suggested the possible implications of ferroptosis in shaping tumor immune microenvironment and immunological features.

## Materials and methods

### Study population and data collection

The current study searched the TNBC cohorts from The Cancer Genome Atlas (TCGA) (https://portal.gdc.cancer.gov/) and GEO (http://www.ncbi.nlm.nih.gov/geo/) together with METABRIC (http://www.cbioportal.org/). The inclusion criteria of TNBC patients comprised the following: i) histopathological diagnosis of TNBC, ii) appropriate transcriptome data, and iii) available follow-up information. As a result, we included 117 patients (tumors, N = 117; normal tissues, N = 113) from TCGA-TNBC as the training cohort. Meanwhile, 107 patients from the GSE58812 (12) and 299 patients from the METABRIC (13) were adopted as the verification cohorts. The specific clinical information is listed in **Supplementary Table 1**.

The ensemble IDs of TCGA-TNBC dataset were mapped to gene symbols in accordance with the annotation of “Homo_sapiens.GRCh38.91.chr.gtf”. Then, gene expression was normalized utilizing the scale approach of the limma package (14), and the mean RNA expression of duplicates was computed, followed by the removal of genes with low abundance. The probes of GSE58812 expression profiles were mapped in line with the GPL570 annotation file, and the mean RNA expression was computed for duplicates. The METABRIC data were acquired from the cBio Cancer Genomics Portal (http://cbioportal.org) (15). Pan-cancer expression profiles were acquired from TCGA project.

### Ferroptosis gene set

Sixty FRGs were collected from previously published literature, which is listed in **Supplementary Table 2**. The position of chromosomes of FRGs was visualized in the Circos plot *via* the RCircos package (16). The levels of FRGs were compared between TNBC and normal tissues.

### Somatic mutation analysis

The somatic variants in Mutation Annotation Format were acquired from the TCGA-TNBC dataset. Overall gene mutation was estimated utilizing the maftools package (17).

### Interaction between ferroptosis-related genes

Functional protein–protein interactions among FRGs were conducted *via* the STRING database (https://string-db.org) (18), which were visualized utilizing Cytoscape software (19).

### Survival analysis

A univariate or multivariate Cox regression approach was adopted to assess the relationships of variables with overall survival (OS) using the survival package. Kaplan–Meier (K-M) curves with the log-rank test were carried out by utilizing the survminer package.

### Functional enrichment analysis

With clusterProfiler package (20), Gene Ontology (GO) together with the Kyoto Encyclopedia of Genes and Genomes (KEGG) pathway enrichment analysis was conducted. False discovery rate (FDR) < 0.05 denoted significant enrichment. The “c2.cp.kegg.v7.2.symbols” gene set was downloaded from Molecular Signatures Database, which was adopted for gene set variation analysis (GSVA) (21). Additionally, the activity of known biological processes was inferred through GSVA. Gene set enrichment analysis (GSEA) was conducted to estimate the significant activity of KEGG pathways between groups (22).

### Consensus clustering

The consensus clustering method from the ConsensusClusterPlus package was employed to infer the number of unsupervised classes across TNBC (23). This analysis was set as agglomerative “k-means” clustering with Euclidean correlation distance, and 80% of the samples were resampled 1,000 times. The discrimination of transcriptome profiling between diverse patterns was displayed through principal component analysis (PCA) by utilizing the limma package. To validate the reproducibility of ferroptosis classification, the unique upregulated markers of each ferroptosis pattern were selected, and sample clustering was implemented in the verification cohorts *via* the NTP algorithm.

### Estimation of immunological features

The relative abundance of tumor-infiltrating immune cells was inferred through the single-sample gene set enrichment analysis (ssGSEA) approach. Tumor purity, immune, and stromal scores were computed *via* the ESTIMATE algorithm (24). RNA expression, methylation, and copy-number alterations of immunomodulators (co-stimulators, co-inhibitors, ligands, receptors, cell adhesion, antigen presentation, and others) were analyzed. Additionally, the activity of common immune checkpoints was computed across TNBC. Seven steps within the cancer-immunity cycle were quantified with ssGSEA in accordance with expression profiling (25).

### Weighted co-expression network analysis

The weighted co-expression network analysis (WGCNA) package (26) was adopted for co-expression analysis. First, an appropriate soft threshold power was chosen to transform the adjacency matrix into the topological overlap matrix. Associations of co-expression modules with ferroptosis classification were computed. The genes in the module that exhibited the strongest relationship with ferroptosis classification were regarded as ferroptosis classification-relevant genes.

### Construction of a ferroptosis scoring system

A least absolute shrinkage and selection operator (LASSO) regression model was conducted on prognostic ferroptosis pattern-relevant genes by utilizing the glmnet package. The coefficient was computed with a multivariate Cox regression approach, and the remaining genes were chosen for constructing a ferroptosis scoring system (ferroptosis_score). Ferroptosis_score was computed as the sum of the products of gene expression and matched coefficients. TNBC cases were classified as ferroptosis_score-high and ferroptosis_score-low groups in accordance with the median score. Time-dependent receiver operating characteristic (ROC) analysis was implemented, followed by calculation of the area under the curve (AUC) at diverse time points for assessing the discriminative significance. Utilizing the rms package, a nomogram was constructed in accordance with prognostic parameters. The predictive efficiency was estimated with calibration curves.

### Evaluation of post-transcriptional events

The miRNA expression profiles were downloaded from TCGA-TNBC dataset. MiRNAs or mRNAs with differential expression were selected between groups in accordance with FDR < 0.05. Targeted mRNAs were inferred through online databases, followed by KEGG pathway enrichment analysis.

### Therapeutic response estimation

The expression similarity between groups and patients receiving anti-PD-1/anti-CTLA4 agents was assessed through Subclass Mapping (SubMap) (27). The chemotherapeutic response was inferred on the basis of the largest publicly available pharmacogenomics database: Genomics of Drug Sensitivity in Cancer (GDSC; www.cancerRxgene.org) (28). The prediction process employed the pRRophetic package, and ridge regression was utilized for estimating half of the maximum inhibitory concentration (IC50) (29). The prediction accuracy was evaluated with 10-fold cross-validation. Drug sensitivity profiling of human cancer cell lines was obtained from the Cancer Therapeutics Response Portal (CTRP) (https://portals.broadinstitute.org/ctrp) together with the PRISM project (https://depmap.org/portal/prism/), which was adopted for the prediction of small-molecule agents (30).

### Statistical analysis

R software (version 4.1.0) was adopted for statistical analysis. Continuous variables were compared with Student’s t-test or Wilcoxon rank-sum test. The relationships between variables were estimated with Pearson’s or Spearman’s test. Statistical significance was set at p < 0.05 (*p < 0.05, **p < 0.01, and ***p < 0.001).

## Results

### Transcriptional and genetic alterations, survival implication, and interactions of ferroptosis-related genes in triple‐negative breast cancer

**Figure 1:**
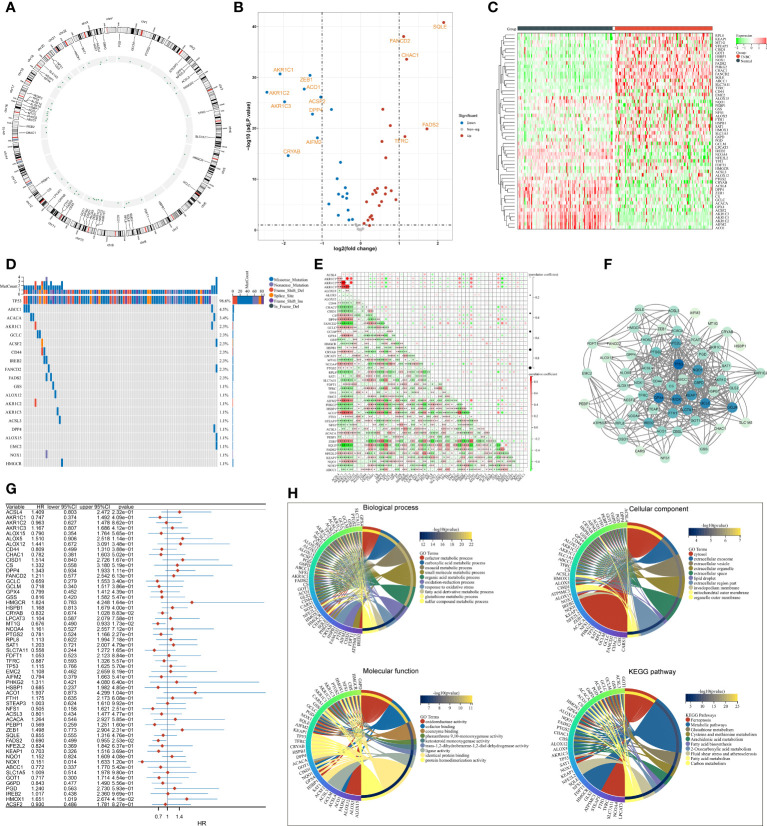
Transcriptional and genetic alterations, survival implication, and interactions of FRGs in TNBC. **(A)** Circos plot for the position of FRGs on chromosomes. **(B, C)** Volcano and heatmap for RNA levels of FRGs in TNBC and normal tissues. **(D)** Oncoplot of the somatic landscape of FRGs across TNBC. FRGs are ranked by mutation frequency, and side bar plot shows log10 converted Q-value estimated through MutSigCV. **(E)** Relationships between FRGs at the RNA level. **(F)** An interaction network of products of FRGs. **(G)** Univariate Cox regression results of the relationships of FRGs with OS. **(H)** GO and KEGG enrichment results of FRGs. FRGs, ferroptosis-related genes; TNBC, triple‐negative breast cancer; OS, overall survival; GO, Gene Ontology; KEGG, Kyoto Encyclopedia of Genes and Genomes *p<0.05, **p<0.01 and ***p<0.001..

The present study collected 60 FRGs from previously published literature. **Figure 1A** illustrates the position of FRGs on chromosomes. Most FRGs exhibited abnormal expression in TNBC in contrast to normal tissues (**Figures 1B, C**). The extensive somatic mutations of FRGs occurred across TNBC, especially TP53 (**Figure 1D**). Pearson’s correlation test of RNA expression unveiled that most FRGs notably interacted with each other (**Figure 1E**). Additionally, we investigated the closely functional interactions among products of FRGs (**Figure 1F**). Among FRGs, MT1G, FADS2, and HMOX1 were notably linked to TNBC cases’ OS (**Figure 1G**). GO and KEGG enrichment results confirmed the implication of FRGs in ferroptosis (**Figure 1H**).

### Definition of ferroptosis classification across triple‐negative breast cancer

**Figure 2:**
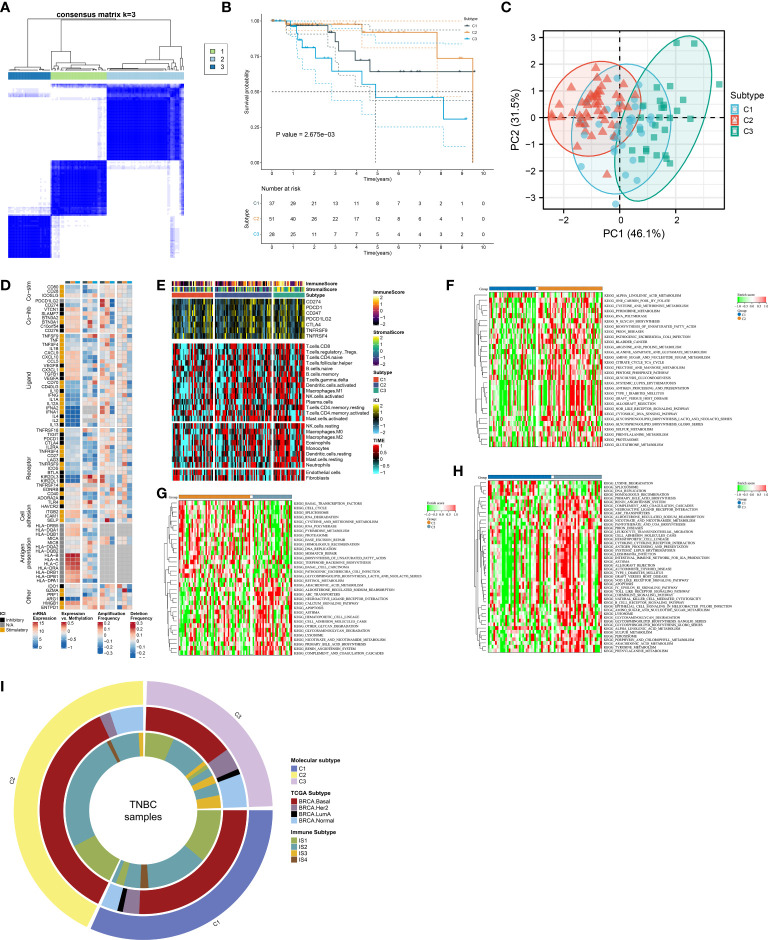
Definition of ferroptosis classification with unique immunological traits in TCGA-TNBC cohort. **(A)** Classification of TNBC patients as three patterns through consensus matrix. **(B)** K-M curves for OS among three ferroptosis patterns. **(C)** PCA plots for the notable discrepancy among ferroptosis patterns. **(D)** Distribution of RNA expression, methylation, and copy-number alterations of immunomodulators in three ferroptosis patterns. **(E)** Distribution of immune checkpoint expression, immune cell abundance, and immune and stromal scores across ferroptosis patterns. **(F–H)** KEGG pathways with different activities in C1 versus C2, C2 versus C3, and C3 versus C1. **(I)** Relationships between the ferroptosis classification with TCGA subtypes and immune subtypes. TNBC, triple‐negative breast cancer; K-M, Kaplan–Meier; OS, overall survival; PCA, principal component analysis; KEGG, Kyoto Encyclopedia of Genes and Genomes; TCGA, The Cancer Genome Atlas.

In accordance with the consensus clustering approach using the transcript levels of prognostic FRGs, the optimal number of clusters was 3, and TCGA-TNBC dataset was categorized as three disparate ferroptosis patterns, namely, C1–3 (**Supplementary Figures 1A–C**; **Figure 2A**). Three patterns exhibited disparate OS results, with C3 being the worst, C1 the next, and C2 the best (**Figure 2B**). PCA unveiled the diverse transcriptome profiling traits among three ferroptosis patterns (**Figure 2C**). We selected the unique upregulated markers in each ferroptosis pattern that were utilized for sample clustering in the GSE58812 and METABRIC datasets (**Supplementary Figure 2A**). The reproducibility of ferroptosis classification was confirmed in the two verification datasets (**Supplementary Figures 2B–E**).

### Unique immunological traits in diverse ferroptosis patterns

The notable discrepancy in RNA expression, methylation, and copy-number variations of immunomodulators (co-stimulators, co-inhibitors, ligands, receptors, cell adhesion, antigen presentation, and others) was observed across three ferroptosis patterns (**Figure 2D**). In addition, the C3 subtype displayed the highest abundance of immune cells and expression of immune checkpoints, followed by C2 and C1 (**Figure 2E**). The activity of KEGG pathways was compared between ferroptosis patterns. In contrast to C1, higher activity of multiple metabolism pathways (pyrimidine, arginine and proline metabolism, alanine aspartate and glutamate, amino sugar and nucleotide sugar, fructose, and mannose, etc.) was observed in C2 (**Figure 2F**). C2 displayed the enhanced activity of biosynthesis and degradation of DNA and RNA than C3 (**Figure 2G**). Additionally, tumorigenic pathways (calcium pathway, apoptosis, ABC transporters, neuroactive ligand–receptor interaction, etc.) possessed increased activity in C3 versus C2 or C1 (**Figures 2G, H**). In contrast to C1, higher activity of immune response pathways (complement and coagulation cascades, antigen processing and presentation, chemokine pathway, etc.) was found in C3 (**Figure 2H**). We compared known molecular subtypes with the ferroptosis classification. Our results demonstrated that the ferroptosis classification was independent of known TCGA subtypes and immune subtypes (**Figure 2I**).

### Selection of ferroptosis pattern-relevant genes in triple‐negative breast cancer

**Figure 3:**
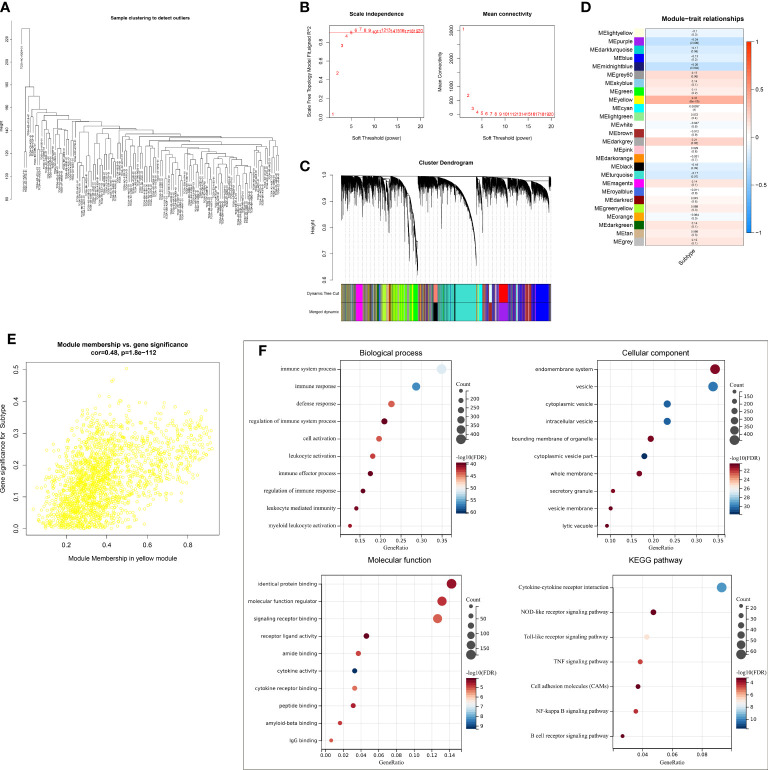
Selection of ferroptosis pattern-relevant genes in TCGA-TNBC cohort. **(A)** Removal of outliers *via* sample clustering. **(B)** Selection of the optimal soft threshold through considering scale independence together with mean connectivity. **(C)** The branches of the clustering dendrogram correspond to 26 modules. **(D)** Relationships of modules with ferroptosis classification. Correlation coefficients and p-values are exhibited in boxes. **(E)** Scatter plots for the correlations of module membership in yellow module with gene significance for ferroptosis classification. **(F)** GO and KEGG enrichment results of ferroptosis pattern-relevant genes. GO, Gene Ontology; KEGG, Kyoto Encyclopedia of Genes and Genomes.

A gene co-expression network was conducted by adopting WGCNA to select modules with the strongest correlation to ferroptosis classification. First, no outliers were detected among TCGA-TNBC samples (**Figure 3A**). The appropriate soft threshold was set as 10 by considering scale independence together with mean connectivity (**Figure 3B**). Afterward, a scale-free co-expression network was established. Consequently, 26 co-expression modules were clustered (**Figure 3C**). Especially, the yellow module displayed the strongest correlation to ferroptosis classification (correlation coefficient = 0.37, p = 6e−05; **Figure 3D**). In addition, module membership in the yellow module was strongly linked to gene significance for ferroptosis classification (**Figure 3E**). Thus, genes in the yellow module were regarded as ferroptosis pattern-relevant genes, which primarily participated in modulating immune response and TNBC-relevant pathways (NOD-like/Toll-like receptor/TNF/NF-κB/B cell receptor pathways, etc.) (**Figure 3F**).

### Establishment and external verification of a robust ferroptosis scoring system in triple‐negative breast cancer

**Figure 4:**
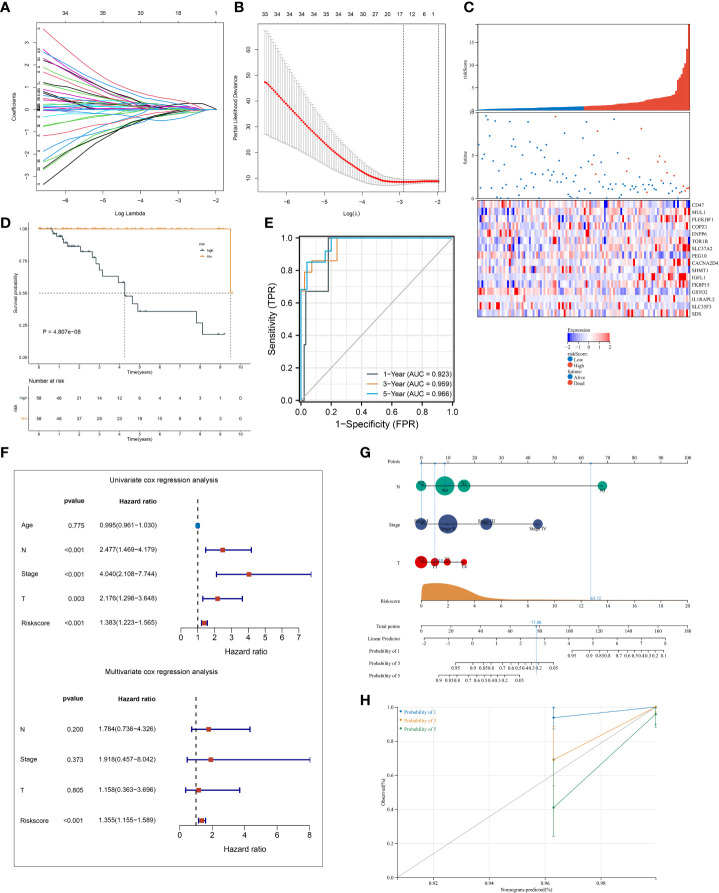
Establishment of a ferroptosis scoring system in TCGA-TNBC cohort. **(A)** Changing trajectory of prognostic ferroptosis pattern-relevant genes. The x-axis denotes the log λ values of independent variables, and the y-axis denotes their coefficients. **(B)** Confidence intervals with distinct λ values. **(C)** Distribution of ferroptosis-relevant risk score, survival status, and gene expression across TNBC cases. **(D)** K-M curves of OS between ferroptosis-relevant high- and low-risk groups. **(E)** ROC curves at 1-, 3- and 5-year OS outcomes. **(F)** Univariate and multivariate Cox regression results of ferroptosis-relevant risk score and clinicopathological parameters with TNBC cases’ OS. **(G)** Construction of the nomogram by totaling the points determined on the points scale for ferroptosis-relevant risk score, T, N, and M stages. **(H)** Calibration curves of the relationships between observed 1-, 3-, and 5-year OS and nomogram-estimated outcomes. TNBC, triple‐negative breast cancer; K-M, Kaplan–Meier; OS, overall survival; ROC, receiver operating characteristic.

A total of 145 ferroptosis pattern-relevant genes notably correlated to TNBC cases’ OS (**Supplementary Table 3**), which were adopted for implementing LASSO regression analysis. Increasing λ led to a decrease in the number of independent variables with coefficients close to 0 (**Figure 4A**). Ten-fold cross-validation was adopted for building the scoring system and estimating the confidence intervals following diverse λ values. The optimal λ value was determined when partial likelihood deviance was the lowest (**Figure 4B**), and 16 genes were selected for the scoring system following the formula: ferroptosis-relevant risk score = (−0.142765874605473) * exp^CD47^ + 0.112505459964827 * exp^MUL1^ + 0.00538161716022841 * exp^PLEKHF1^ + 0.215213236835648 * exp^COPZ1^ + (−0.168998590096113) * exp^ENPP6^ + 0.0791918658709983 * exp^TOR1B^ + 0.254809882047955 * exp^SLC37A2^ + (−0.0946087366980265) * exp^PEG10^ + 0.139388903981419 * exp^CACNA2D4^ + (−0.0109640256571299) * exp^SHMT1^ + 0.385024690538088 * exp^IGFL1^ + 0.0181238671012704 * exp^FKBP15^ + (−0.373571481244596) * exp^GSTO2^ + 0.113747451219178 * exp^IL1RAPL2^ + (−0.0758300765619583) * exp^SLC35F3^ + 0.0576459041484211 * exp^SDS^. The risk score of each case in TCGA-TNBC cohort was computed, and cases were classified as ferroptosis-relevant high- and low-risk groups under the median score (**Figure 4C**). K-M curves showed poorer OS outcomes in the high-risk score group (**Figure 4D**). ROC analysis was conducted on the ferroptosis-relevant scoring system for estimating prognostic outcomes. The AUCs at 1-, 3-, and 5-year OS were all >0.9 (**Figure 4E**), demonstrating the excellent efficiency of the scoring system in prognostic estimation in TCGA-TNBC cohort. The robustness of the ferroptosis-relevant scoring system was proven in GSE58812 and METABRIC cohorts, and the same formula was applied to the two verification cohorts. Notable differences were observed in OS between ferroptosis-relevant high- and low-risk groups, with relatively high AUCs at 1-, 3-, and 5-year OS in GSE58812 and METABRIC cohorts (**Supplementary Figures 3A–F**). The above data demonstrated that the ferroptosis-relevant scoring system possessed favorable robustness on diverse platforms.

Univariate Cox regression results showed that T, N, and M stages and the ferroptosis-relevant risk score were significantly associated with TNBC OS (**Figure 4F**). Further multivariate Cox regression analysis demonstrated that the ferroptosis-relevant risk score acted as an independent risk parameter of TNBC prognosis (**Figure 4F**). To assist in the clinical application of the ferroptosis-relevant scoring system, we proposed a prognostic nomogram by incorporating T, N, and M stages and the ferroptosis-relevant risk score (**Figure 4G**). Calibration curves confirmed the high accuracy of the nomogram in inferring 1-, 3-, and 5-year OS outcomes (**Figure 4H**).

### Ferroptosis-relevant risk score involved in somatic mutation and transcriptional and post-transcriptional mechanisms

**Figure 5:**
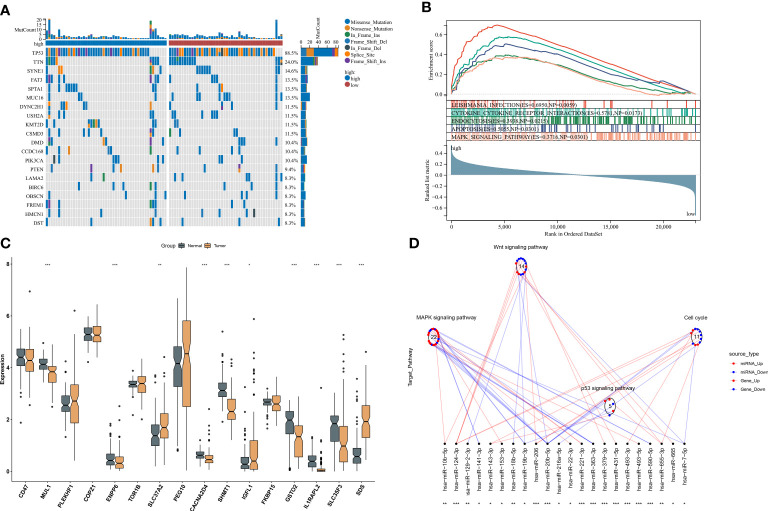
Ferroptosis-relevant risk score involved in somatic mutation and transcriptional and post-transcriptional mechanisms in TCGA-TNBC cohort. **(A)** Oncoplot for the somatic landscape of ferroptosis-relevant high- and low-risk patients. Genes are ranked by mutation frequency. **(B)** GSEA for the activity differences in KEGG pathways between groups. **(C)** Levels of the genes from ferroptosis-relevant risk score in TNBC and normal tissues. **(D)** Differences in miRNA-targeted signaling pathways between groups. Red dots denote miRNA-targeted genes that exhibit upregulation in the high-risk group, while blue dots denote miRNA-targeted genes that exhibit downregulation. Red lines indicate low expression of miRNAs in the high-risk group, while blue lines indicate highly expressed miRNAs in the low-risk group. The circle denotes a signaling pathway enriched by targeted genes. GSEA, gene set enrichment analysis; KEGG, Kyoto Encyclopedia of Genes and Genomes; TNBC, triple‐negative breast cancer. *p < 0.05, **p < 0.01 and ***p < 0.001.

**Figure 6:**
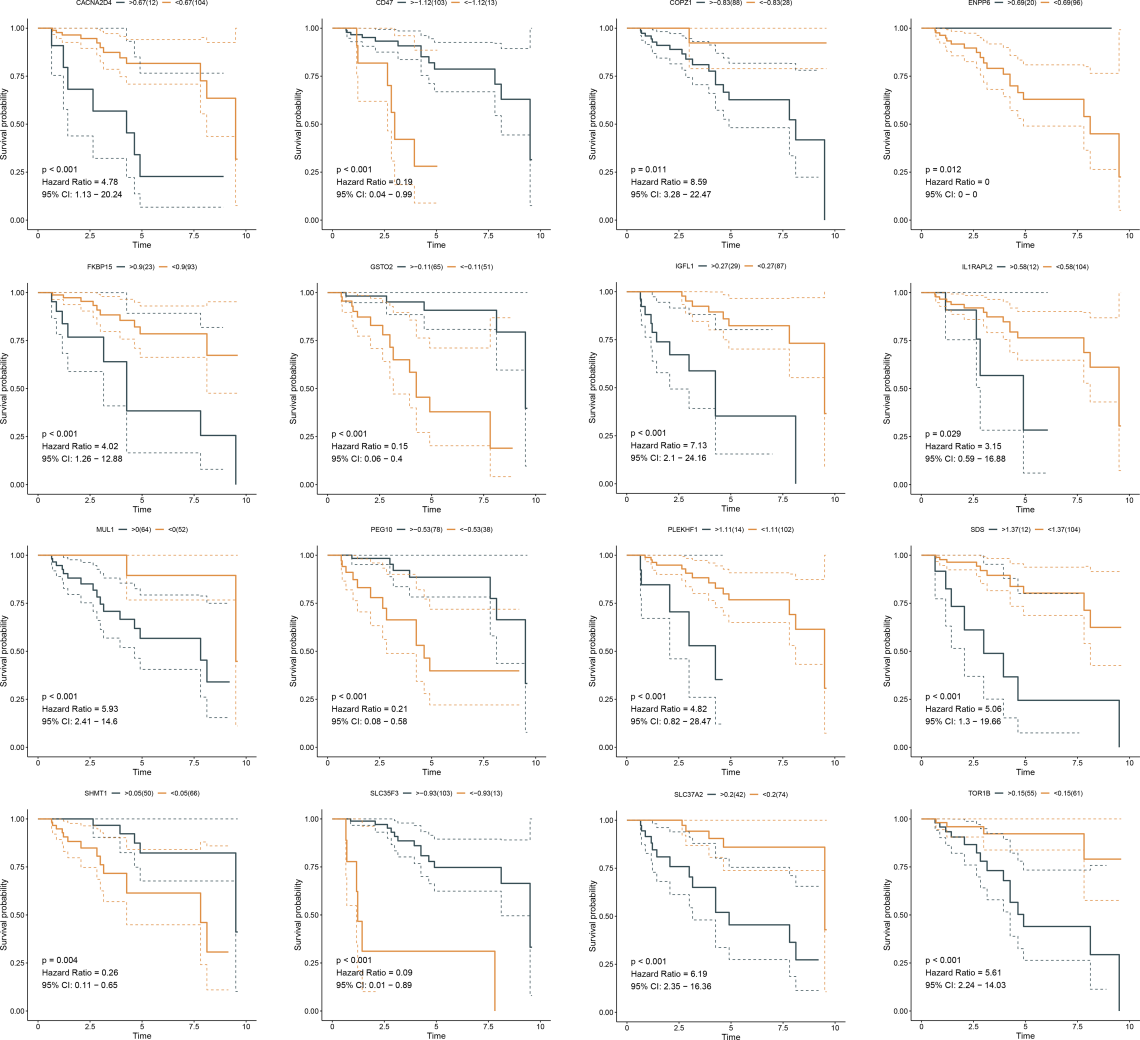
K-M curves exhibit OS outcomes in the high and low expression of genes from ferroptosis-relevant risk scores across TCGA-TNBC patients. K-M, Kaplan–Meier; OS, overall survival.

Somatic mutation was compared between ferroptosis-relevant high- and low-risk groups. **Figure 5A** illustrates the top 20 mutated genes across TCGA-TNBC individuals, with TP53 as the most frequent mutated gene. A higher mutation frequency was observed in low-risk populations. Additionally, *Leishmania* infection, cytokine–cytokine receptor interaction, endocytosis, apoptosis, and MAPK signaling pathway displayed prominently increased activity in high-risk cases in contrast to low-risk cases (**Figure 5B**). Most genes from the ferroptosis-relevant risk score displayed abnormal expression between TNBC and normal tissues, with upregulated SLC37A2, IGFL1, and SDS and downregulated MUL1, ENPP6, CACNA2D4, SHMT1, GSTO2, IL1RAPL2, and SLC35F3 (**Figure 5C**). Afterward, the present study assessed differences in miRNA expression between ferroptosis-relevant high- and low-risk groups in TCGA-TNBC dataset. A total of 84 miRNAs notably differentially expressed between groups were selected (**Supplementary Table 4**). Moreover, an enrichment analysis of their target genes was implemented. The miRNA target genes exhibited observable correlations to cell cycle, MAPK, Wnt, and p53 signaling pathways, which were differentially expressed between groups (**Figure 5D**), suggesting that the ferroptosis-relevant risk score might correlate to miRNA expression and signaling pathway activity. The survival significance of each gene in the ferroptosis-relevant risk score was assessed. In **Figure 6**, upregulated CACNA2D4, COPZ1, FKBP15, IGFL1, IL1RAPL2, MUL1, PLEKHF1, SDS, SLC37A2, and TOR1B were correlated to poorer OS outcomes, with opposite effects of CD47, ENPP6, GSTO2, PEG10, SHMT1, and SLC35F3 on OS.

### Immunological traits and the immunotherapeutic response of distinct ferroptosis-relevant risk score groups

**Figure 7:**
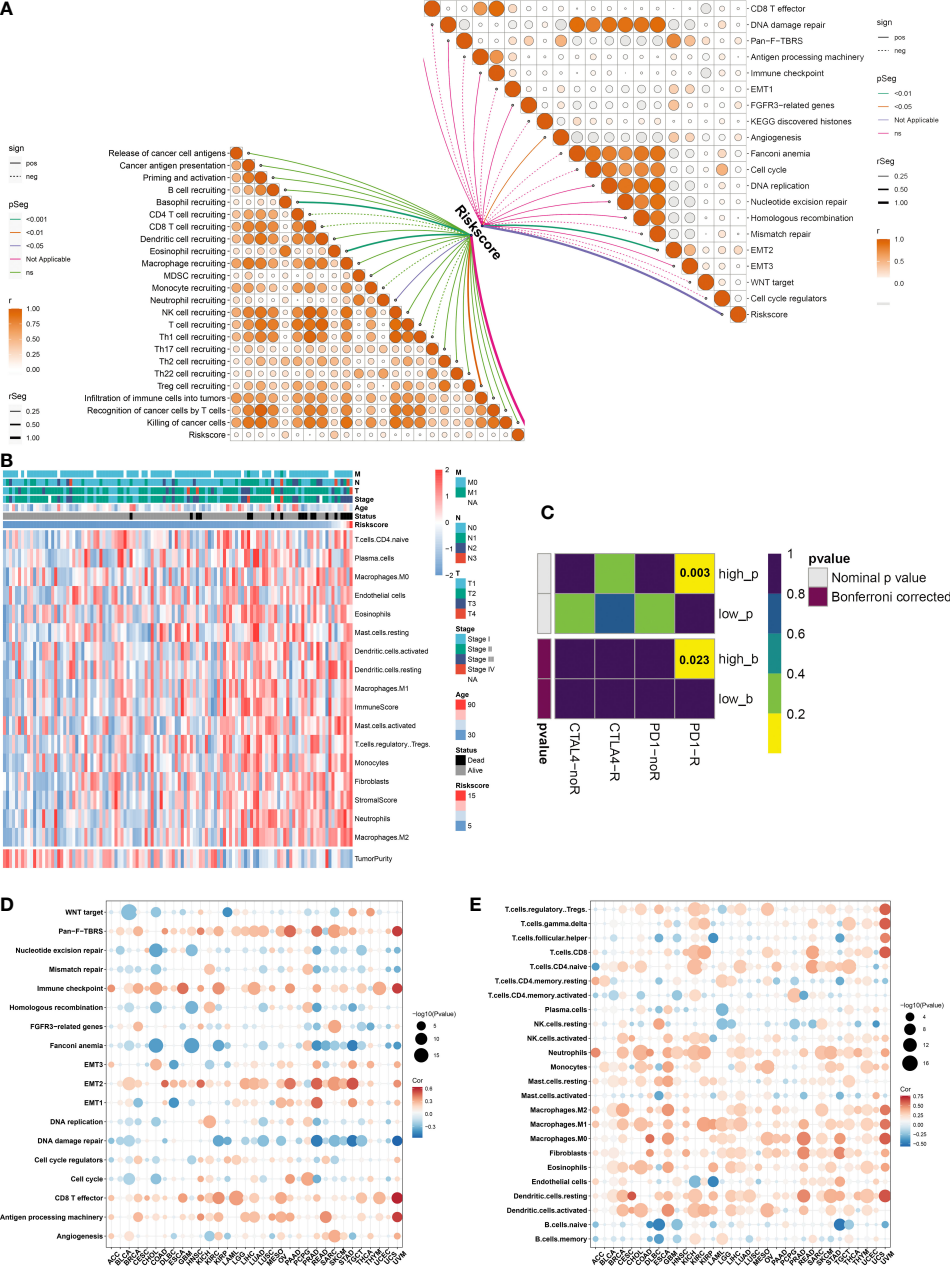
Immunological traits and immunotherapeutic response of distinct ferroptosis-relevant risk score groups. **(A)** Relationships between risk score and activity of each step within cancer-immunity cycle and known biological processes in TCGA-TNBC cohort. **(B)** Distribution of immune cell infiltrations, tumor purity, and immune and stromal scores across TCGA-TNBC patients. **(C)** Estimation of immunotherapeutic response of high- and low-risk TCGA-TNBC individuals through SubMap analysis. **(D, E)** Pan-cancer analysis of the relationships between risk score and activity of known biological processes and abundance of immune cell types. SubMap, Subclass Mapping.

The activity of steps within the cancer-immunity cycle was computed, which may reflect an anti-tumor immune response. We observed that the ferroptosis-relevant risk score was positively linked to most steps (**Figure 7A**). In addition, this risk score displayed notably positive interactions with angiogenesis and EMT2. A higher abundance of most immune cells, increased immune and stromal scores, and decreased tumor purity were observed in the high-risk group (**Figure 7B**). By adopting the SubMap algorithm, the therapeutic response of high- and low-risk cases was inferred. Consequently, high-risk cases possessed a higher possibility of benefitting from anti-PD-1 immunotherapy (**Figure 7C**).

### Immune relevance of ferroptosis-relevant risk score across pan-cancer

Pan-cancer analysis was implemented to further elucidate the immune relevance of the ferroptosis-relevant risk score. This risk score exhibited generally positive relationships with the immune checkpoint, CD8 T effector, and antigen-processing machinery in most cancer types (**Figure 7D**). In addition, this risk score was positively linked to the abundance of most immune cell types across pan-cancer (**Figure 7E**). The above data demonstrated the crucial roles of the ferroptosis-relevant risk score in the tumor immune microenvironment across pan-cancer.

### Potential therapeutic significance of ferroptosis-relevant gene signature

**Figure 8:**
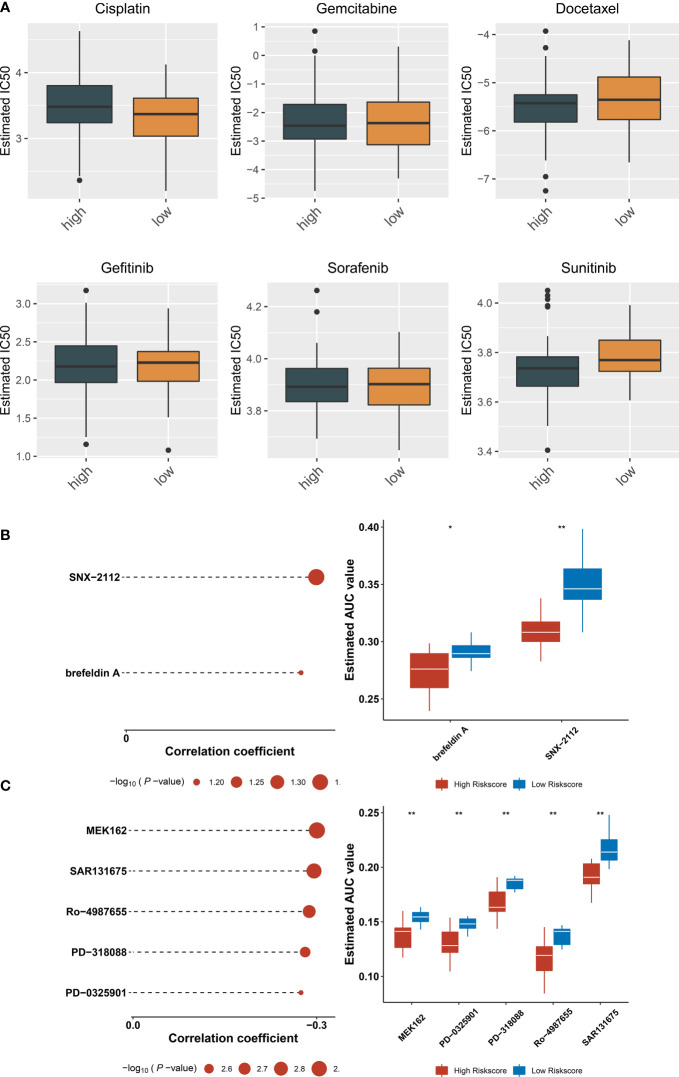
Potential therapeutic significance of ferroptosis-relevant gene signature in TCGA-TNBC cohort. **(A)** Comparison of IC50 values of chemotherapy or targeted therapeutic agents between ferroptosis-relevant high- and low-risk TNBC cases. **(B)** Association between CTRP-derived agents and ferroptosis-relevant risk score and comparison of AUC values of agents between groups. **(C)** Association between PRISM-derived agents and ferroptosis-relevant risk score and comparison of AUC values of agents between groups. TNBC, triple‐negative breast cancer; CTRP, Cancer Therapeutics Response Portal; AUC, area under the curve. *p < 0.05, **p < 0.01.

Estimated IC50 values of chemotherapy or targeted therapeutic agents were compared between ferroptosis-relevant high- and low-risk TNBC cases. Data showed that the low-risk group exhibited significantly lower IC50 of cisplatin, with significantly lower IC50 of sunitinib in the high-risk group (**Figure 8A**), indicating that low-risk cases had higher sensitivity to cisplatin, with higher sensitivity to sunitinib for high-risk cases. Potential small-molecule agents were predicted aiming at high-risk cases. As a result, two CTRP-derived agents (SNX-2112 and brefeldin A; **Figure 8B**) and PRISM-derived agents (MEK162, PD-0325901, PD-318088, Ro-4987655, and SAR131675; **Figure 8C**) were selected for high-risk patients.

## Discussion

TNBC is the most aggressive subtype of breast carcinoma, with higher recurrent risk and mortality compared with other subtypes. Due to limited therapeutic options, it is urgently required for ascertaining therapeutic agents with a unique mode of action for surmounting current issues in TNBC therapy (31). Ferroptosis is an iron-dependent cell death form with the traits of accumulated reactive oxygen species together with lipid peroxidation products. Targeting ferroptosis is regarded as a novel anti-TNBC strategy (32). Inducing ferroptosis may sensitize TNBC cells to radiotherapy (33) and neoadjuvant chemotherapy, etc. (34). Nonetheless, FRGs have been rarely studied in patients with TNBC.

On the basis of the expression profiling of prognostic FRGs, a novel ferroptosis classification was proposed for TNBC. Each ferroptosis pattern possessed unique prognostic and immunological traits. Evidence demonstrates the interactions of ferroptosis with tumor immunity. For instance, the interplay between TNBC cells and macrophages modulates ferroptotic cell death, development, and chemoresistance of TNBC (35). Ferroptosis of cancer cells negatively affects antigen-presenting cells and impedes adaptive immune responses, thereby hindering ferroptosis-induced therapeutic application (36). The LASSO approach is a compressed estimate adopted for acquiring a refined model through building a penalty function, which enables to compress several coefficients and set several coefficients to 0 (37). Hence, this approach reserves the preponderance of gene subset choice with shrinkage and is a biased estimator that is appropriate for dealing with complex collinear data, thus helping select variables in parameter estimation for better solving the multicollinearity issue of regression analysis. Based on ferroptosis pattern-relevant genes, a robust ferroptosis scoring system in inferring TNBC survival was proposed. High- and low-risk groups exhibited diverse somatic mutation and transcriptional and post-transcriptional mechanisms. The activation of innate immunity and tumoral ferroptotic cell death may induce anti-PD-1/PD-L1 therapeutic resistance (38). High-risk cases were more likely to benefit from anti-PD-1 immunotherapy.

Neoadjuvant chemotherapy remains a crucial treatment option for patients with locally advanced TNBC, which lowers tumor burden, offers the opportunity for surgery and even breast conservation, and accelerates early evaluation of individual responses (34). Evidence demonstrates that patients achieving pathological complete responses (pCRs) following neoadjuvant chemotherapy have more favorable survival (39). Nevertheless, currently, pCRs remain in low proportions. Improving pCRs represents an important aim of neoadjuvant chemotherapy. Thus, early detection of cases with better responses to neoadjuvant chemotherapy has naturally become the major focus of TNBC therapy. The ferroptosis-relevant gene signature may infer the sensitivity of TNBC to cisplatin and sunitinib. Low-risk cases had higher sensitivity to cisplatin, and high-risk cases were more sensitive to sunitinib. Small-molecule agents exert crucial roles in cancer therapy, combining with specific target molecules in cells for playing specific functions. They have become the focus of research because of their potent specificity, prominent curative effect, and little damage to normal cells. We predicted two CTRP-derived agents (SNX-2112 and brefeldin A) and PRISM-derived agents (MEK162, PD-0325901, PD-318088, Ro-4987655, and SAR131675) for the treatment of high-risk patients.

In summary, our study reported the prognostic, molecular, immunological, and pharmacogenomic features linked to ferroptotic cell death in TNBC. As our awareness of ferroptosis continues to improve, we look forward to more research to unveil the potential mechanisms of ferroptosis in cancer.

## Conclusion

Altogether, our findings proposed three disparate ferroptosis patterns across TNBC, which provided a novel insight into the relationships of ferroptosis with prognostic outcomes and immunological features. Additionally, a robust ferroptosis-relevant gene signature was exploited, which enabled us to precisely speculate survival and responses to immunotherapy, chemotherapy, and targeted therapies for TNBC individuals. Several small-molecule agents were also predicted based on ferroptosis-relevant gene signatures. Hence, this study provided a roadmap for patients’ stratification and assisted in developing regimens for personalized treatment decisions and follow-up in TNBC.

## Data availability statement

The original contributions presented in the study are included in the article/**Supplementary Material**. Further inquiries can be directed to the corresponding author.

## Author contributions

KF conceived and designed the study. ZX and SJ conducted most of the experiments and data analysis, and wrote the manuscript. DT, CY, and YH participated in collecting data and helped to draft the manuscript. All authors contributed to the article and approved the submitted version.

## Funding

The research was supported by a Ningxia Hui Autonomous Region Natural Science Foundation Project (2022AAC03748), Ningxia Hui Autonomous Region Natural Science Foundation Project (2021AAC03523), Basic research project of Yinchuan Maternal and Child Health Hospital (2022NYFYCX05).

## Conflict of interest

The authors declare that the research was conducted in the absence of any commercial or financial relationships that could be construed as a potential conflict of interest.

## Publisher’s note

All claims expressed in this article are solely those of the authors and do not necessarily represent those of their affiliated organizations, or those of the publisher, the editors and the reviewers. Any product that may be evaluated in this article, or claim that may be made by its manufacturer, is not guaranteed or endorsed by the publisher.

## Supplementary material

Supplementary Figure 1 Consensus clustering analysis of TCGA-TNBC samples. **(A)** Consensus cumulative distribution function (CDF) curve. **(B)** Relative alterations in the area under the CDF curve. **(C)** Tracking plot of sample classification.

Supplementary Figure 2 Verification of reproducibility of ferroptosis classification. **(A)** Heatmap illustrates the unique up-regulated markers in each ferroptosis pattern across TCGA-TNBC samples. **(B)** Sample clustering of the GSE58812 dataset through NTP algorithm based on the unique up-regulated markers. **(C)** K-M curves of OS among three clusters in the GSE58812 dataset. **(D)** Sample clustering of the METABRIC dataset with NTP algorithm on the basis of the unique up-regulated markers. **(E)** K-M curves of OS among three clusters in the METABRIC dataset.

Supplementary Figure 3 External validation of the robustness of ferroptosis-relevant gene signature. **(A)** Distribution of ferroptosis_score, survival status, and gene expression in the GSE58812 dataset. **(B)** K-M curves of OS between ferroptosis-relevant high- and low-risk groups in the GSE58812 dataset. **(C)** ROC curves at 1-, 3- and 5-year OS in the GSE58812 dataset. **(D)** Distribution of ferroptosis-relevant risk score, survival status, and gene expression in the METABRIC dataset. **(E)** K-M curves of OS between ferroptosis-relevant high- and low-risk groups in the METABRIC dataset. **(F)** ROC curves at 1-, 3- and 5-year OS in the METABRIC dataset.

Supplementary Table 1 The clinical information of TNBC patients from TCGA-TNBC, GSE58812, and METABRIC cohorts.

Supplementary Table 2 The list of ferroptosis-related genes.

Supplementary Table 3 Univariate cox regression results of ferroptosis pattern-relevant genes.

Supplementary Table 4 Differentially expressed miRNAs between ferroptosis-relevant high- and low-risk groups in the TCGA-TNBC dataset.

## Abbreviations

TNBC, triple-negative breast cancer; ICB, immune checkpoint blockade; FRGs, ferroptosis-related genes; K-M, Kaplan–Meier; OS, overall survival; GO, Gene Ontology; KEGG, Kyoto Encyclopedia of Genes and Genomes; FDR, false discovery rate; GSVA, gene set variation analysis; GSEA, gene set enrichment analysis; WGCNA, weighted co-expression network analysis; LASSO, least absolute shrinkage and selection operator; ROC, receiver operating characteristic; AUC, area under the curve; SubMap, Subclass Mapping; GDSC, Genomics of Drug Sensitivity in Cancer.
