# Supplementary figures and images for: Integrated profiling uncovers prognostic, immunological, and pharmacogenomic features of ferroptosis in triple-negative breast cancer
Source: Front Immunol. 2022 Nov 25;13:985861. doi: 10.3389/fimmu.2022.985861 (PMC9732280; doi:10.3389/fimmu.2022.985861)

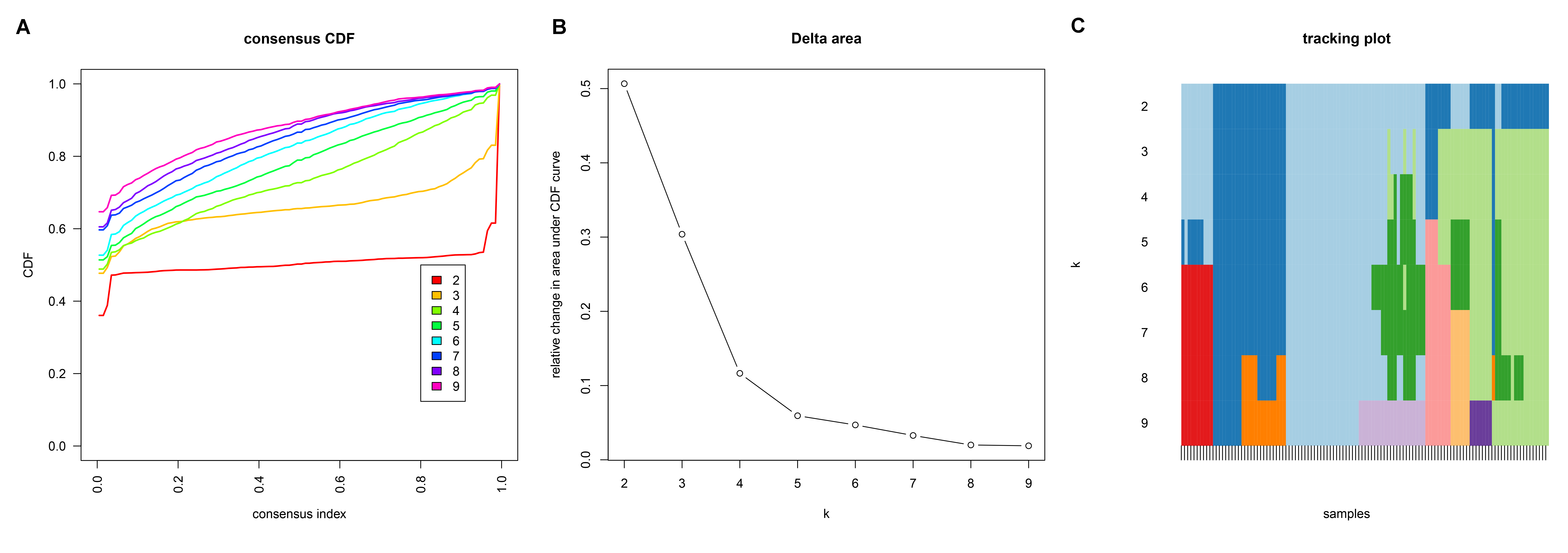

Supplement: Supplementary Figure 1 — Consensus clustering analysis of TCGA-TNBC samples. (A) Consensus cumulative distribution function (CDF) curve. (B) Relative alterations in the area under the CDF curve. (C) Tracking plot of sample classification. [file Image_1.tif]

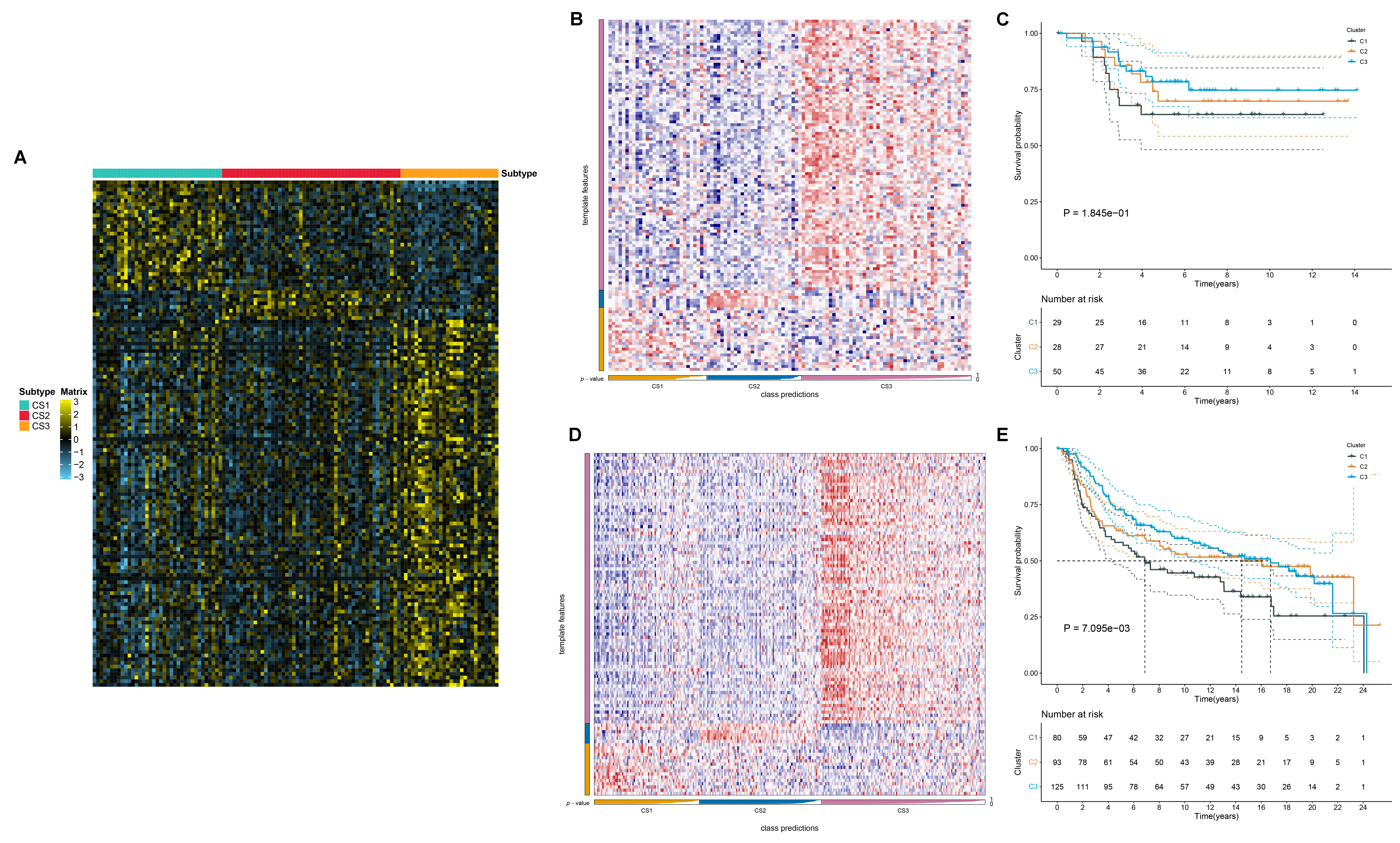

Supplement: Supplementary Figure 2 — Verification of reproducibility of ferroptosis classification. (A) Heatmap illustrates the unique up-regulated markers in each ferroptosis pattern across TCGA-TNBC samples. (B) Sample clustering of the GSE58812 dataset through NTP algorithm based on the unique up-regulated markers. (C) K-M curves of OS among three clusters in the GSE58812 dataset. (D) Sample clustering of the METABRIC dataset with NTP algorithm on the basis of the unique up-regulated markers. (E) K-M curves of OS among three clusters in the METABRIC dataset. [file Image_2.tif]

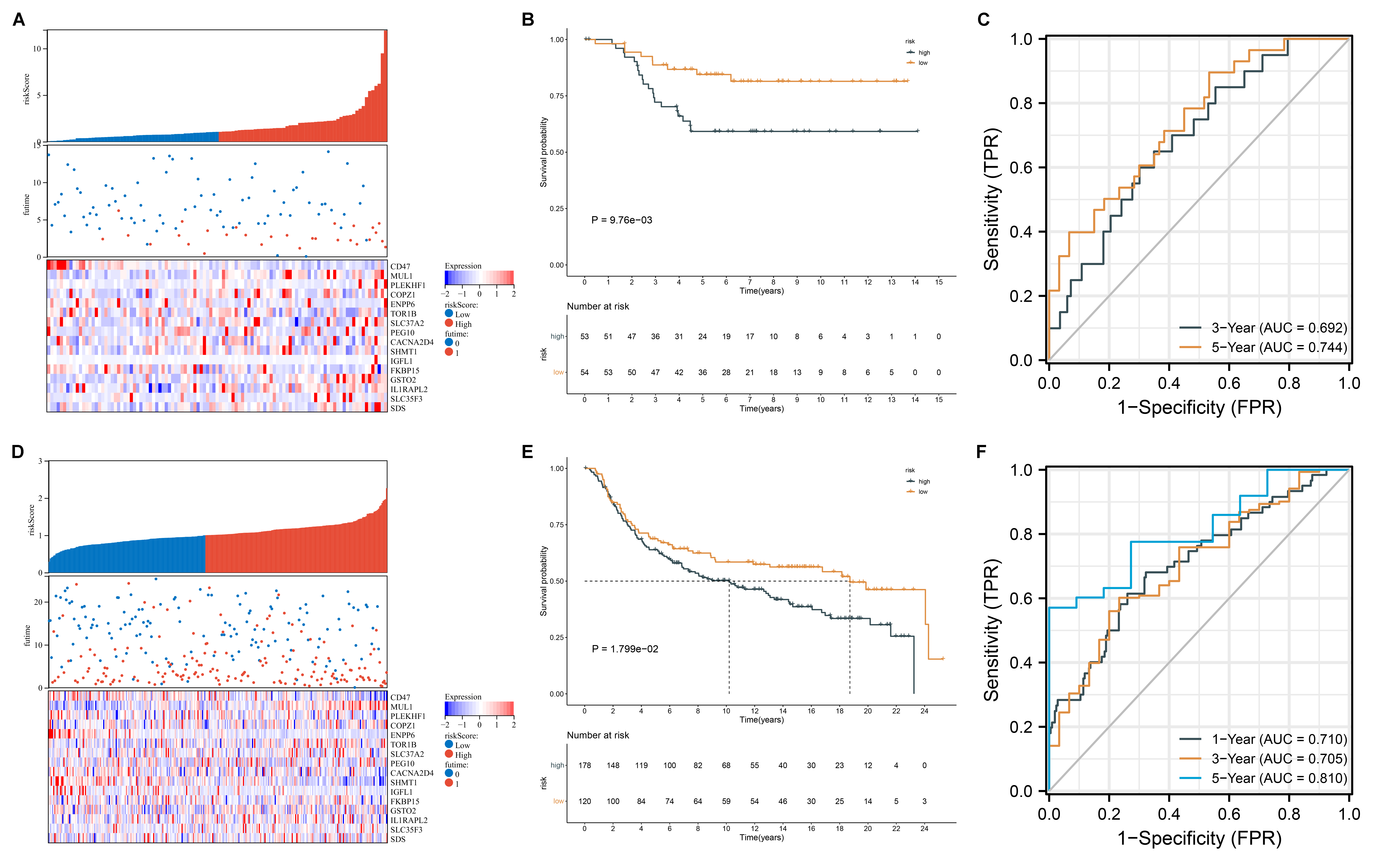

Supplement: Supplementary Figure 3 — External validation of the robustness of ferroptosis-relevant gene signature. (A) Distribution of ferroptosis_score, survival status, and gene expression in the GSE58812 dataset. (B) K-M curves of OS between ferroptosis-relevant high- and low-risk groups in the GSE58812 dataset. (C) ROC curves at 1-, 3- and 5-year OS in the GSE58812 dataset. (D) Distribution of ferroptosis-relevant risk score, survival status, and gene expression in the METABRIC dataset. (E) K-M curves of OS between ferroptosis-relevant high- and low-risk groups in the METABRIC dataset. (F) ROC curves at 1-, 3- and 5-year OS in the METABRIC dataset. [file Image_3.tif]
